# Identification of a *Pm4* Allele as a Powdery Mildew Resistance Gene in Wheat Line Xiaomaomai

**DOI:** 10.3390/ijms23031194

**Published:** 2022-01-21

**Authors:** Danyu Yao, Waqas Ijaz, Yi Liu, Jinghuang Hu, Wentao Peng, Bowen Zhang, Xiaolan Wen, Juan Wang, Dan Qiu, Hongjie Li, Shihe Xiao, Guozhong Sun

**Affiliations:** National Engineering Research Center of Crop Molecular Breeding, Institute of Crop Science, Chinese Academy of Agricultural Sciences, Beijing 100081, China; yaodanyu@caas.cn (D.Y.); Waqas.surani@yahoo.com (W.I.); LY2668174837@163.com (Y.L.); hujh2016@163.com (J.H.); pengwentao5298@163.com (W.P.); zhangbw19980329@163.com (B.Z.); wxl992021@163.com (X.W.); wj_1714661899@163.com (J.W.); 18518757886@163.com (D.Q.); lihongjie@caas.cn (H.L.)

**Keywords:** *Triticum aestivum*, powdery mildew, BSR-Seq, SNP, *Pm4*, haplotype

## Abstract

Powdery mildew, caused by *Blumeria graminis* f. sp. *tritici* (*Bgt*), is one of the most destructive foliar diseases of wheat. In this study, we combined the bulked segregant RNA sequencing (BSR-seq) and comparative genomics analysis to localize the powdery mildew resistance gene in Chinese landrace Xiaomaomai. Genetic analysis of F_1_ plants from a crossing of Xiaomaomai × Lumai23 and the derived F_2_ population suggests that a single recessive gene, designated as *pmXMM*, confers the resistance in this germplasm. A genetic linkage map was constructed using the newly developed SNP markers and *pmXMM* was mapped to the distal end of chromosome 2AL. The two flanking markers *2AL15* and *2AL34* were closely linked to *pmXMM* at the genetic distance of 3.9 cM and 1.4 cM, respectively. Using the diagnostic primers of *Pm4*, we confirmed that Xiaomaomai carries a *Pm4* allele and the gene function was further validated by the virus-induced gene silencing (VIGS). In addition, we systematically analyzed *pmXMM* in comparison with the other *Pm4* alleles. The results suggest that *pmXMM* is identical to *Pm4d* and *Pm4e* at sequence level. *Pm4b* is also not different from *Pm4c* according to their genome/amino acid sequences. Only a few nucleotide variances were detected between *pmXMM* and *Pm4a/b*, which indicate the haplotype variation of the *Pm4* gene.

## 1. Introduction

Wheat (*Triticum aestivum* L.) is one of the most widely grown food crops worldwide. Its stable yield plays an important role in food security. Powdery mildew, caused by *Blumeria graminis* f. sp. *tritici* (*Bgt*), is one of the most devastating foliar diseases of wheat. At present, the most efficient and environmentally safe approach to counteract epidemics of powdery mildew is to breed disease-resistant cultivars in production. However, most of powdery mildew resistance (*R*) genes are race-specific which confer strong immunity to some but not all of the pathogen races. Therefore, molecular identification and utilization of new *R* genes to produce a wheat cultivar with improved disease resistance is challenging for wheat breeders.

To date, more than 60 loci for resistance to powdery mildew (*Pm1*–*Pm66*) have been documented [1] and over ten genes have been cloned in wheat landraces [2,3,4,5,6,7]. Most of the identified *R* genes in crops encode proteins with predicted coiled-coil-nucleotide-binding-site (CC-NBS) and leucine-rich repeat (LRR) domains [8,9]. The exception was *Pm24*, which encodes a tandem kinase [10]. *Stpk-V*, a key member of *Pm21*, was demonstrated to be a serine/threonine kinase [11]. *Pm38*/*Lr34*/*Yr18*/*Sr57* and *Pm26*/*Yr46*/*Lr67*/*Sr55*, which, respectively, provides broad-spectrum resistance to multiple pathogens and encodes an ABC transporter and hexose transporter [12,13]. In addition, *Pm4*, a resistance gene valuable for the breeding, was recently cloned and illustrated to be a putative serine/threonine kinase [14,15].

Multiple *Pm4* alleles located on chromosome 2AL have been reported. *Pm4a* and *Pm4b* originated from *T. dicoccum* and *T. carthlicum*, respectively, and can be found in many commercial cultivars in China [14,16,17]. Schmolke et al. (2012) identified a dominant gene *Pm4d*, which was introduced from *T. monococcum.* In addition, the two alleles *Pm4c* and *Pm4e* were detected in common wheat cultivars [18,19]. Besides the *Pm4* locus, few other genes were also mapped to chromosome 2AL and demonstrated to be allelic or closely linked to *Pm4*, such as *PmPS5A* [20], *pmLK906* [21], *PmHNK54* [22], *pmX* [23], and *Pm50* [24]. Therefore, they have been considered to be members of the *Pm4* locus.

Next-generation sequencing offers a new genetic mapping strategy that combines bulked segregant analysis (BSA) with RNA-sequencing analysis for quick identification of wheat powdery mildew resistance genes [25]. Molecular markers developed from single nucleotide polymorphisms (SNP) enable the establishment of a high-resolution genetic linkage map and detection of the target genes. In this study, we have identified a recessive powdery mildew resistance gene from Chinese wheat landrace Xiaomaomai employing this technique. The constructed linkage map localized the resistance gene, designated as *pmXMM*, to chromosome 2AL, flanked by two newly designed SNP markers *2AL15* and *2AL34*. We further confirmed that *pmXMM* was a haplotype of *Pm4* showing an identical genome sequence to *Pm4d* and *Pm4e*. The functionality of *pmXMM* was further verified by the virus-induced gene silencing. This study suggests that *Pm4* is a powdery mildew resistance gene valuable for developing future disease-resistant wheat cultivars.

## 2. Materials and Method

### 2.1. Plant Materials

Bread wheat cultivar Xiaomaomai is a Chinese landrace and shows resistance to powdery mildew. The susceptible recurrent parent Lumai23 is a Chinese winter wheat cultivar widely grown in the Shandong province. F_1_, F_2_, and F_2:3_ resistance segregation populations were created by crossing Xiaomaomai with Lumai23 and subsequently used for the genetic analysis and molecular mapping. Common wheat lines Khapli/8*Cc (carrying gene *Pm4a*), Armada (carrying gene *Pm4b*), 81-7241 (carrying gene *Pm4c*), and D29 (carrying gene *Pm4c*) were used in resistance spectrum and genetic analyses. Winter wheat cultivar Zhongzuo9504 was used as the susceptible control to assess the powdery mildew resistance.

### 2.2. Resistance Evaluation

The conidiospores of isolate *Bgt1* were freshly increased on the susceptible cultivar Zhongzuo9504. When abundant sporulation was visible on the leaf area of Zhongzuo9504, the inoculation was conducted using the dusting technique: one-leaf-stage seedlings were sprayed with water evenly on the leaf area and then dusted with conidiospores of *Bgt1*. One pot of Zhongzuo9504 was used to inoculate at most five pots of tested seedlings. After inoculation, plants were grown in a growth chamber maintained at 16 °C, 16-h light, 8-h dark, and 50% humidity. After 7–10 days of inoculation, when the susceptible control Zhongzuo9504 plants were heavily diseased, the disease symptom of each plant was visually rated on a 0–4 scale as described by Liu et al. (1999). At least 15 plants were tested for each F_2:3_ family. Plants were considered as resistant when the infection types (IT) were 0–2, while considered susceptible when the scores were 3–4. The effectiveness of *pmXMM* in wheat line Xiaomaomai against powdery mildew was tested using 40 *Bgt* isolates derived from single-spore isolation collected from different wheat fields located in Hebei, Beijing, Tianjin, Shandong, Henan, Sichuan, and Shanxi of China (Supplementary Table S1).

### 2.3. Genotyping of F_2:3_ Lines Using BSR-Seq Analysis

The phenotypically contrasting F_2:3_ families against isolate *Bgt1* were used to construct the resistant and susceptible RNA pools (respectively made of six homozygous resistant and homozygous susceptible F_2:3_ families) for RNA-seq analysis. Total RNA of the two bulks of leaf samples were separately extracted using the RNAsimple Total RNA Kit (Tiangen). RNA-Seq was done by the platform of Illumina HiSeq4000 (Beijing Southern Genome Research Technology Co., Ltd., Beijing, China). The raw sequencing reads were quality controlled using software Trimmomatic v0.36 [26] with the default parameters. Using software STAR v2.5.1b [27], the clean reads were aligned to the wheat reference genome assembly IWGSC RefSeq v1.0 [28] with the mismatch rate of less than 5%. The uniquely mapped read pairs were used in further analysis. The read alignments were masked for PCR duplications before they were used to call SNPs and InDels using small variant caller Strelka v2 [29]. The resulting SNPs and InDels with sequencing depth less than 6 were discarded, and the remaining ones were applied to bulked segregant analysis. Only variants with allele frequency difference (AFD) > 0.8 and *p*-value of Fisher’s exact test on read count data < 1 × 10^−10^ were classified as trait-associated variants and used as templates for marker development.

### 2.4. Marker Analysis

Genomic DNA was extracted from the leaf tissue of wheat seedlings using a DNAquik Plant System (Tiangen). The single nucleotide polymorphisms (SNPs) associated with the powdery mildew resistance were identified by BSR-Seq analysis and selected for genome-specific Kompetitive Allele Specific PCR (KASP) markers development using the Polymarker website (http://www.polymarker.info/ accessed on September 2020). PCR was performed in a 10 μL reaction mixture using 2 × KASP Master Mix (Std Rox, LGC) following the protocol. Polymorphic KASP markers between the parents and the contrasting DNA bulks were then used to construct the genetic linkage map of *pmXMM*. Polymorphism survey was also conducted with SSR markers linked to *Pm4* locus on chromosome 2AL [18,19,23,30,31,32] (Supplementary Table S2). PCR was performed with a C1000 Touch^TM^ Thermal Cycler (BIORAD) in a 10 μL reaction mixture using the 2 × M5 PAGE Taq PCR Mix (Mei5bio) according to the manufacturer’s instructions. The program condition was 95 °C for 3 min, 35 cycles of 94 °C for 25 s, 55 °C for 25 s, and 72 °C for 20 s, and a final extension at 72 °C for 5 min. PCR products were separated in 8% non-denaturing polyacrylamide gels with a 19:1 or 39:1 acrylamide/bisacrylamide ratio, and then silver-stained as described by Santos et al. (1993). The dCAP markers was developed to determine the SNPs of *Pm4* alleles. A total of 443 bp fragments were first amplified using the primer pair *Pm4.3* listed in Supplementary Table S3 and subjected to one-hour digestion at 37 °C using NeaI enzyme. Products were subsequently investigated on the 2% agarose gel.

### 2.5. Data Analysis and Linkage Map Construction

The Chi-squared test (χ^2^) was employed to examine whether the observed separation data in F_2_ and F_2:3_ population fit for Mendelian segregation ratio. The genetic distance between the polymorphic markers and the target gene was calculated by the Kosambi function and the genetic linkage map was constructed using software Mapdraw v2.1 [33]. A logarithm of the odd ratio (LOD) of 3.0 was used as the threshold for declaration of linkage and the maximum genetic distance allowed between markers was set at 50.0 cM.

### 2.6. BSMV Virus-Induced Gene Silencing

To prepare the recombinant BSMV:*Pm4-V2* constructs, two different fragments (200–500 bp) amplified from *Pm4* exons 6 and 7 were respectively subcloned into the pBS-BSMV-γ vector using a One Step Seamless Cloning kit (GeneBetter) according to the manufacturer’s instructions (Primer sequences were listed in Supplementary Table S3). For in vivo synthesis of viral RNA, an equimolar amount of pBS-BSMV-α, pBS-BSMV-β, and pBS-BSMV-γ vectors were first transformed into the one-month-old tobacco plants mediated by *Agrobacterium*. Ten days after injection, tobacco leaves with virus symptom were ground and subsequently used to rubbing the second leaf of Xiaomaomai plants at the two-leaf stage. Seedlings of the same age were inoculated with the wild-type (γ0) virus as a control. Fourteen days after virus infection, the third leaves of Xiaomaomai were collected for qRT-PCR analysis and the fourth leaves were infected with the isolate *Bgt1*. Powdery mildew phenotype was documented 10 days post inoculation.

### 2.7. RNA Isolation and RT-PCR

The third leaf of wheat seedling was harvested 14 days post inoculation of virus and ground in liquid nitrogen. RNA was extracted using the TRIzol^®^ reagent (Biorigin) according to the manufacturer’s instructions. The FastKing gDNA Dispelling RT SuperMix kit (Tiangen) was employed to remove residual DNA and synthesize the corresponding cDNA using the following PCR program: 42 °C for 15 min and 95 °C for 3 min.

### 2.8. Real-Time Quantitative PCR

Real-time PCR was used to determine the transcript abundance of *Pm4* gene. Samples were run in triplicate with PerfectStart Green qPCR SuperMix (TransGen Biotech) on a Bio-Rad CFX96 Touch Real-Time PCR Detection System. The wheat *actin* gene was used as a housekeeping control and qRT-PCR primers used for the target and reference genes are shown in Supplementary Table S3. The thermocycling conditions were 94 °C for the 30 s, followed by 44 cycles of 94 °C for 5 s, 58 °C for 15 s, and 72 °C for 10 s. Relative quantities were calculated and normalized to the reference genes using 2^−^^ΔΔ^^Ct^ method [34].

## 3. Results

### 3.1. Resistance of Xiaomaomai to Different Bgt Isolates

Forty *Bgt* isolates collected from the northern part of China were used to examine the virulence spectrum of Xiaomaomai together with the wheat cultivars carrying *Pm4a*, *Pm4b*, and *Pm4c* (Supplementary Table S1). Xiaomaomai exhibited resistance to 12 isolates (30%, IT 0 to 2) and susceptibility to 28 isolates (IT 3 or 4) shown in Supplementary Table S1. Xiaomaomai and the wheat lines carrying *Pm4* alleles revealed similar reaction patterns: line Khapli/8*Cc containing *Pm4a* was resistant to 30% of the isolates tested and 81-7241 carrying *Pm4c* was resistant to 32.5% of them. Line Armada containing *Pm4b* was effectively against to 37.5% isolates and exhibited different reaction to three isolates compared to Xiaomaomai. Zhongzuo9504 was employed as a susceptible control.

### 3.2. Inheritance of the Powdery Mildew Resistance in Xiaomaomai

**Figure 1:**
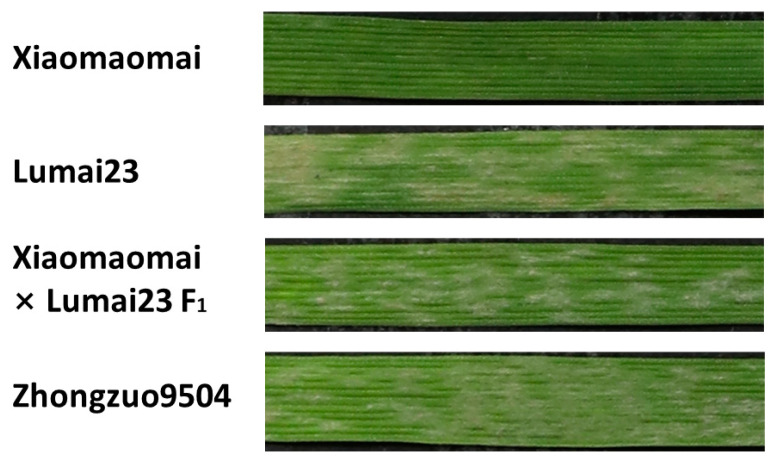
The phenotypic reactions of resistant parent Xiaomaomai, susceptible parent Lumai23, and their F_1_ progenies to *Bgt1* isolate. Zhongzuo9504 served as the susceptible control.

Xiaomaomai and Lumai23 reacted differently when the seedlings were inoculated with *Bgt1* isolate from Hebei province (Figure 1). Therefore, this isolate was used for phenotypic analysis on F_1_, F_2_, and F_2:3_ populations derived from Xiaomaomai × Lumai23 cross. All the F_1_ plants showed susceptible to *Bgt1*, while F_2_ plants exhibited a segregation ratio of 1:3 for resistant and susceptible plants (χ^2^_1:3_ = 0.581, *p* = 0.446, Table 1). In the progeny test, a segregation ratio of 1:2:1 (homozygous resistant lines: heterozygous lines: homozygous susceptible lines) was observed in the F_2:3_ population, consisting of 177 families (χ^2^ _1:2:1_ = 0.831, *p* = 0.660). This result indicated that a single recessive gene, tentatively designated as *pmXMM*, confers the resistance to isolate *Bgt1* in Xiaomaomai.

### 3.3. RNA-Seq Analysis of the RNA Bulks with Distinct Reactions to Bgt1

**Figure 2:**
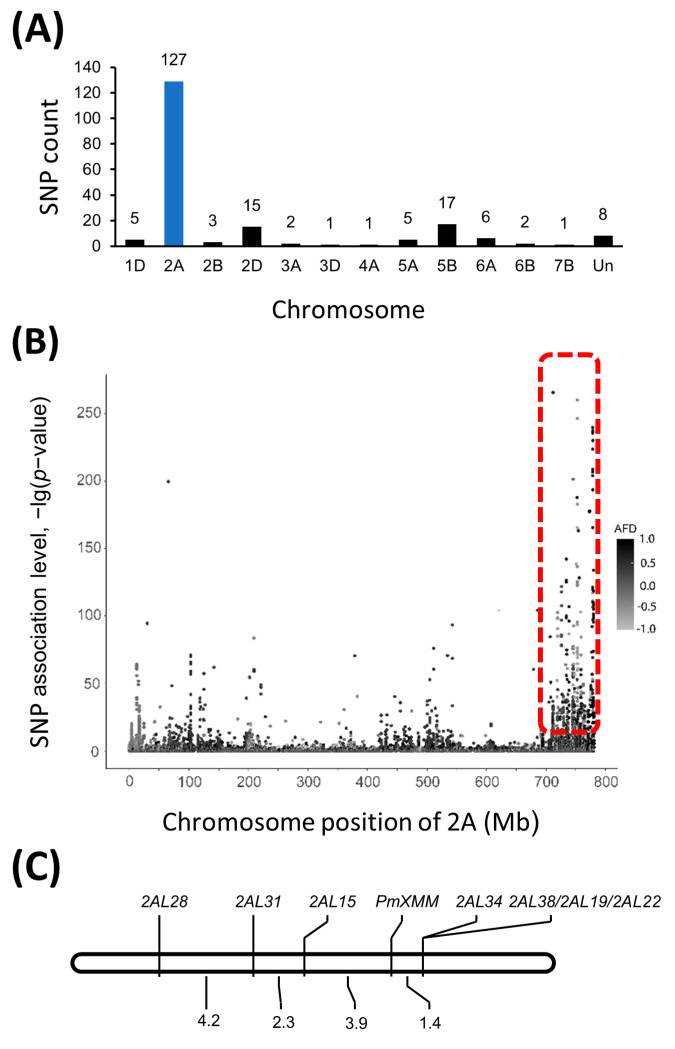
BSR-Seq analysis and genetic linkage map of *pmXMM*. The number of single nucleotide polymorphisms (SNPs) distributed on different wheat chromosomes (**A**) and the SNP variants on chromosome 2A (**B**). Linkage map of *pmXMM* on chromosome 2AL (**C**).

The RNA samples pooled from F_2:3_ families with known *Bgt1* resistance and susceptibility were designated as Bulk-R and Bulk-S, respectively. Through RNA-Seq analysis performed on an Illumina HiSeq 4000 platform, 39,574,081 and 40,826,755 raw read pairs were created for Bulk-R and Bulk-S. After quality control, 36,902,452 read pairs of Bulk-R were selected and 31,793,499 (86.16%) of them were uniquely mapped to the wheat reference genome assembly IWGSC RefSeq v1.0 [28]. In addition, there were 39,313,629 high-quality reads and 35,409,901 (90.07%) uniquely mapped reads for the Bulk-S sample. A total of 328,746 variants were identified from those mapped reads by the Strelka software [29] with default parameters, of which 195 variants were found to be trait-associated (*p* < 1 × 10^−10^ and AFD > 0.8). We found that 127 trait-associated variants were enriched in a 54 Mb genomic interval (727,191,545–780,717,753) on chromosome arm 2AL in the Chinese Spring reference genome (Figure 2A,B, Supplementary Table S4), suggesting that the target gene was potentially localized in this region.

### 3.4. Polymorphic Analysis of SNP Markers and Construction of Genetic Linkage Map

To further map the resistance gene, polymorphism survey was initially conducted with previously reported markers linked to *Pm4* locus, which was also located on chromosome 2AL. The assay was carried out between the crossing parents as well as the two contrasting DNA bulks made of F_2:3_-resistant and -susceptible progenies. The surveyed markers were shown in Supplementary Table S2 and only *Pm4b*-associated maker *STS470* was polymorphic between the two parents and contrasting DNA bulks. We subsequently developed the SNP markers flanking sequences of the SNPs potentially associated with the target gene on chromosome 2AL. Seven pairs of primers were shown to be able to differentiate the crossing parents (Supplementary Table S5). Consistent polymorphism was also detected between the DNA bulks containing resistant and susceptible progenies, indicating that they were likely linked to *pmXMM*. Therefore, these SNP markers were employed for the construction of a genetic linkage map using 355 F_2:3_ families derived from Xiaomaomai × Lumai23 cross. Results suggested that *2AL15*, *2AL28*, and *2AL31* were potentially mapped on the proximal side, while *2AL22*, *2AL19*, *2AL38*, and *2AL34* were localized on the distal side of the target gene (Table 2, Figure 2C). *pmXMM* was localized in 3.7 Mb physical region (755,705,200–759,456,729) on chromosome 2AL flanked by markers *2AL15* and *2AL34* with genetic distances of 1.4 and 3.9 cM, respectively (Figure 2C).

### 3.5. Identification of the pmXMM Candidate Gene

**Figure 3:**
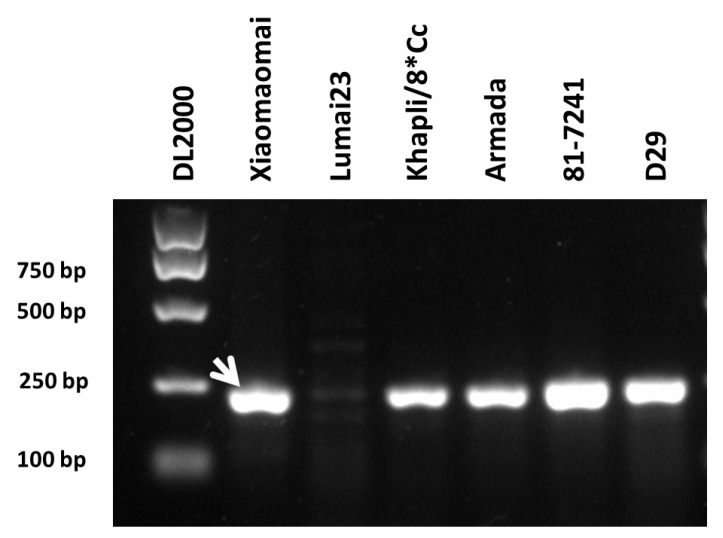
Amplification pattern of *Pm4*-specific marker *Pm4.1* amplified from the genomic DNA of Xiaomaomai, Lumai23, and wheat lines carrying *Pm4a* (Khapli/8*Cc), *Pm4b* (Armada), *Pm4c* (81-7241), and *Pm4e* (D29) in 1% agarose gel. The first lane was loaded with DL2000 DNA ladder. The white arrow indicates the amplicons specific for the *Pm4* gene.

**Figure 4:**
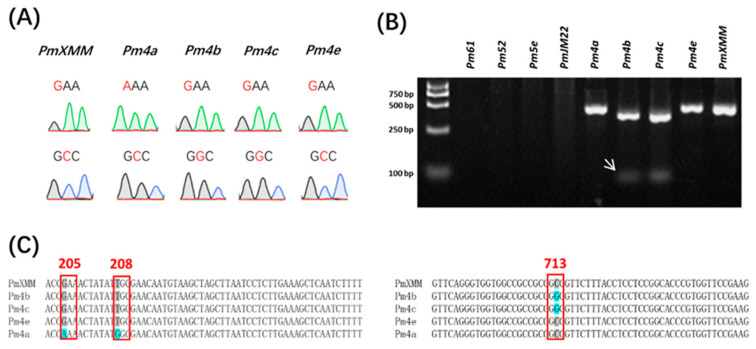
Profiles of designed SNP markers differentiating *pmXMM*, *Pm4a*, *Pm4b*, *Pm4c*, and *Pm4e*. Sanger sequencing profiles of SNP markers targeting the 205 (Up) and 713 (Bottom) amino acid sites of *Pm4* alleles (**A**). Polymorphic profile of wheat lines showing resistance to *Bgt 1* isolate using the dCAP marker (**B**). White arrows indicate the polymorphic bands specific for *Pm4b* and *Pm4c*. No fragment was detected in wheat lines carrying *Pm61*, *Pm52*, *Pm5e*, and *PmJM22*. The first lane was loaded with a DL2000 DNA ladder. Sequence alignments of investigated *Pm4* alleles at the 205, 208, and 713 amino acid variant sites (**C**). Conserved nucleotides are indicated by a grey color and the variant sites causing amino acid changes are highlighted by a blue color.

*Pm4* gene mapped to the chromosomal region similar to *pmXMM* has been identified recently (Sánchez-Martín et al., 2021). It encodes a putative chimeric protein of a serine/threonine kinase and has two splicing variants (*Pm4-V1* and *Pm4-V2*). By sequencing the exons of *Pm4*, Sánchez-Martín et al. (2021) have shown that the four amino acid polymorphisms listed in Table 3 represents the variation of Pm4-resistant proteins they have checked. To determine the allelic relationship of *pmXMM* to *Pm4*, we employed a molecular marker *Pm4.1* designed from the *Pm4* coding sequence to investigate the genetic background of the Xiaomaomai and Lumai23 (Supplementary Table S3). The presence of *Pm4* alleles was detected in Xiaomaomai and the other *Pm4* carrying lines, but not in susceptible parent line Lumai23 (Figure 3). In addition, linkage analysis illustrated that the *Pm4*-specific marker was co-segregating with the target gene (Table 2), suggesting that the resistance gene was likely to be an allele of *Pm4*. We further amplify the exon fragments of *Pm4* alleles, covering the amino acid variances from the genomic DNA of wheat lines carrying *pmXMM*, *Pm4a*, *Pm4b*, *Pm4c*, and *Pm4e* using the primers listed in Supplementary Table S3 and their protein sequences were identified based on the two alternatively spliced transcripts of *Pm4b* (GenBank accession numbers of *Pm4b_V1* CDS and *Pm4b_V2* CDS were MT783929 and MT783930). By Sanger sequencing, we could differentiate *pmXMM* from the other *Pm4* alleles by checking the SNPs at the 205 and 713 amino acid sites (Figure 4A,C). In addition, we have developed a dCAP marker that could be used for the genetic analysis of SNP at the 713 amino acid site of *Pm4* (Figure 4B). However, wheat lines carrying other *Pm* genes and showing resistance to *Bgt 1* isolate were detected blank on the 2% agarose gel using the same dCAP marker (Figure 4B). These results revealed that the investigated *Pm4* genes were divided into three haplotypes: *pmXMM* exhibited the identical genome/amino acid sequence to *Pm4e* and *Pm4d* (Table 3, Sánchez-Martín et al., 2021). *Pm4b* seems to be as same as *Pm4c* according to their genome/amino acid sequence. Only a few nucleotide differences exist between *pmXMM* with *Pm4a*/*b* (Table 3).

### 3.6. Silencing of pmXMM Using BSMV-VIGS

**Figure 5:**
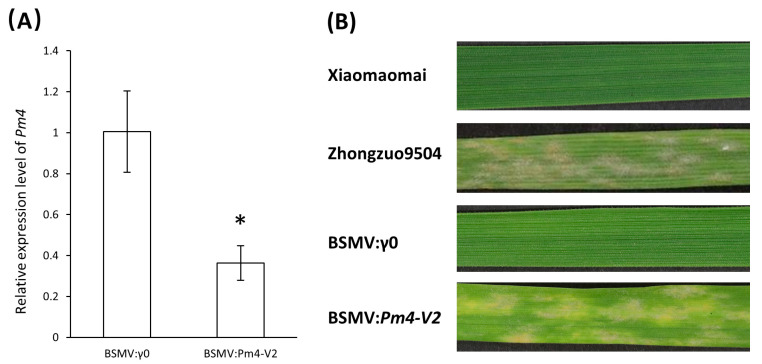
Validation of *pmXMM* functionality by BSMV-VIGS. qRT-PCR analysis of plants infected with BSMV:γ0 and BSMV:*Pm4-V2* virus (**A**). Data are mean ± SE calculated from three biological replicates and normalized to the *actin* expression level. Asterisk indicates a significant difference (*p*-value < 0.05) using Student’s *t*-test. Symptoms of the fourth leaves pre-inoculated with BSMV and then challenged with *Bgt1* isolate (**B**).

To verify the function of *pmXMM* in resistance to *Bgt1* infection in Xiaomaomai, we performed the virus-induced gene silencing (VIGS) as developed with barley stripe mosaic virus (BSMV). Two constructs targeting *Pm4-V2* variance were used to suppress the *pmXMM* expression in Xiaomaomai plants (Supplementary Table S3). Results of real-time quantitative PCR showed that BSMV:*Pm4-V2* virus led to the downregulation of *Pm4* transcript 14 days post inoculation (Figure 5A). Sporulating mildew colonies were observed covering large leaf areas of Xiaomaomai infected with BMSV:*Pm4-V2* virus, suggesting that silencing of *pmXMM* resulted in the susceptibility of Xiaomaomai to *Bgt1* isolate (Figure 5B). Wheat plants infected with wild-type virus BSMV:γ0 served as the control for all the analyses.

## 4. Discussion

This study was designed to locate the candidate gene, conferring the resistance of powdery mildew in Chinese landrace Xiaomaomai. BSR-Seq analysis demonstrated that *pmXMM*, a recessive powdery mildew resistance gene, was located on the distal end of chromosome arm 2AL. Newly developed SNP markers mapped *pmXMM* gene in a 5.3 cM genetic interval corresponding to 3.7 Mb genomic region. Diagnostic primers and Sanger sequencing suggested that Xiaomaomai carries a *Pm4* allele identical to *Pm4d* and *Pm4e* in genome sequences. In addition, the functionality of *pmXMM* in powdery mildew resistance was ultimately confirmed by the virus-induced gene silencing. Thus, we conclude that *pmXMM* is a recessive allele of the *Pm4* locus.

*Pm4* is used in disease resistance breeding because of its effectiveness in the resistance of *Bgt* isolates in certain regions of China and the United States [35,36]. In this study, the frequency of *pmXMM* in 90 wheat cultivars was evaluated (Supplementary Table S6). The results showed that *pmXMM* was present in eight wheat lines, including Huacheng 3366, Zhengyumai 518, Xinong 16, Zhongluo 08-1, Zhengmai 101, Jingnong 3668, 10BMY12, and Lankao 815. In the most recent study, *Pm4* has been cloned by MutChromSeq and was demonstrated to encode a putative serine/threonine kinase [15]. Up to now, over ten wheat powdery mildew resistance genes have been cloned in wheat, including *Pm2* [2], *Pm3b* [3], *Pm5* [4], *Pm8*/*Pm17* [5,37], *Pm21* [7,38], *Pm24* [10], *Pm38*/*Lr34*/*Yr18*/*Sr57* [12], *Pm41* [39], *Pm60* [6], and *Pm46*/*Lr67*/*Yr46*/*Sr55* [13]. *Stpk-V*, located on the *Pm21* locus, was also a serine/threonine kinase [11]. Several disease resistance genes have been identified to contain a serine/threonine kinase domain, such as the stem rust resistance gene *Rpg5* in barley and stripe rust resistance gene *Yr36* in wheat [40,41].

*Pm4* locus including multiple resistance alleles was previously reported to be located on chromosome 2AL (McIntosh and Bennett, 1979). Several *Pm4* allelic genes have been identified to be dominant, including *Pm4a* (Ma et al., 2004), *Pm4b* [32,42], *Pm4c/Pm23* (Hao et al., 2008), *Pm4d* (Schmolke et al., 2012), *Pm4e* (Li et al., 2017), and *PmHNK54* (Xu et al., 2011). *pmXMM* showed the same genome/amino acid sequence as *Pm4d* and *Pm4e* (Table 3), suggesting that these three alleles were likely to be the same haplotype. However, we confirmed that *pmXMM* was a recessive gene by progeny analysis using F_1_ plants developed from the crossing of Xiaomaomai × Lumai23 and the derived F_2_ and F_2:3_ populations (Table 1). One explanation could be that the genomic background of Xiaomaomai was very different from the plant lines carrying *Pm4d* and *Pm4e*. The lack of polymorphism between Xiaomaomai and Lumai23 using common markers linked to *Pm4* supports our speculation. Therefore, only two copies of the gene could lead to the resistance of *Bgt* isolates in Xiaomaomai. Consistent with our results, *pmX* (Fu et al., 2013) and *pmLK906* [43] have been reported to be a recessive gene close to the chromosomal position of *Pm4* alleles. Further research could be carried out to verify whether these two genes were new alleles of *Pm4* using the diagnostic primers and Sanger sequencing.

In the present study, based on the BSR-Seq analysis, SNP markers were designed and used for the construction of the *pmXMM* linkage map (Figure 2C). *pmXMM* was located in chromosome 2AL and spanned a physical interval of about 3.7 Mb (755,705,200–759,456,729) on the Chinese Spring. However, a previous study demonstrated that *Pm4e* was placed in a physical interval of 762.5–768.0 Mb on chromosome 2AL through a fine mapping [44]. Consistently, Sánchez-Martín et al. (2021) reported that *Pm4a/b* was absent in Chinese Spring and the closest homology is located approximately at position 761 Mb. It seems that the chromosomal location of *Pm4* genes in different wheat lines is close but still a little bit different. Similar phenomenon was also observed in the study of Fu et al. (2013), who showed that the common markers exhibited different genetic distances to the same *Pm4* haplotype. For instance, it has been shown that STS marker *XresPm4* was 6.5 cM from *Pm4e* but was cosegregating with *Pm4d* in previous studies [19,31]. In our work, most of the tested *Pm4* associated markers failed to produce the polymorphic banding pattern between the crossing parents as well as the contrasting progeny bulks. Therefore, we speculated that an interstitial missing or inversion might have occurred in wheat plants, which could cause the variation of gene location referring to the Chinese spring genome. Other factors may be the map population and the different genetic background of the crossing parents.

Sánchez-Martín et al. (2021) have shown that *Pm4* was a relatively widespread gene and the polymorphism in a single amino acid could determine the functionality of the protein. For instance, they have discovered three new *Pm4* alleles (*Pm4f*, *Pm4g*, and *Pm4h*) by screening the genetic background of 512 wheat collections. Although there was a single nucleotide change, *Pm4f* carrying lines were susceptible to the tested isolate, while plants containing *Pm4h* showed powdery mildew resistance. In support of this claim, Xiaomaomai showed differential patterns of response to *Bgt* isolates compared to plant lines carrying *Pm4a*, *Pm4b*, and *Pm4c* (Supplementary Table S1). This may be due to the fact that few amino acid variances on the kinase domain or transmembrane domain are crucial for affecting the kinase activity. Consistently, it has been reported that the deletion of the specific two amino acids in the kinase I domain of *Pm24* determined its resistance function [10]. In addition, *Lr67*, a multi-pathogen resistant gene that encodes a predicted hexose transporter, is different from its susceptible form by two amino acids [13]. Other factors, including the genetic background of a plant line, gene-gene interaction, host-pathogen interaction, and environmental conditions, may also contribute to the infection type of a wheat line.

In this study, we designed a new pair of primers that could specifically amplify 213 bp fragment of the *Pm4* gene and produce the diagnostic banding pattern more efficiently (Figure 3). This was beneficial for marker-assisted breeding and stacking *Pm4* with other *Pm* genes to improve the powdery mildew resistance of wheat.

## Figures and Tables

**Table 1:** Progeny test of Xiaomaomai × Lumai23 cross in term of response to *Blumeria graminis* f. sp. *tritici* isolate *Bgt1*.

Parents/ Cross	Generation	Total Numbers of Plants/Families	Phenotype and Number of the Tested F_2:3_ Families			Expected Ratio	χ^2^	*p*-Value
			Resistant	Segregating	Susceptible			
Xiaomaomai	Pr	20	20					
Lumai23	Ps	20			20			
Xiaomaomai × Lumai23	F_1_	25			25			
	F_2_	186	51		135	1:3	0.581	0.446
	F_2:3_	177	46	92	39	1:2:1	0.831	0.660

Pr and Ps indicate a resistant and susceptible parent, respectively.

**Table 2:** Newly developed single nucleotide polymorphism (SNP) markers linked to *pmXMM*.

**F_2:3_ Family Lines**		**SNP Markers**			**Phenotype**	***Pm4-*** **Specific Marker *Pm4.1***	**SNP Markers**		
	*2AL22*	*2AL19*	*2AL38*	*2AL34*			*2AL15*	*2AL31*	*2AL28*
63	B	B	B	B	B	B	B	B	B
158	A	A	A	A	A	A/H	A	A	A
87	A	A	A	A	A	A/H	A	A	**H**
55	B	B	B	B	B	B	B	B	**H**
143	B	B	B	B	B	B	B	B	**H**
144	A	A	A	A	A	A/H	A	A	**H**
100	A	A	A	A	A	A/H	A	A	**H**
111	A	A	A	A	A	A/H	A	**H**	**H**
62	A	A	A	A	A	A/H	A	**H**	**H**
171	B	B	B	B	B	B	B	**H**	**H**
78	A	A	A	A	A	A/H	**H**	**H**	**H**
127	A	A	A	A	A	A/H	**H**	**H**	**H**
51	B	B	B	B	B	B	**H**	**H**	**H**
40	**A**	**A**	**A**	**A**	H	A/H	H	H	H
141	**A**	**A**	**A**	**A**	H	A/H	H	H	H
160	**H**	**H**	**H**	**H**	A	A/H	A	A	A
79	**B**	**B**	**B**	**B**	H	A/H	H	H	H
123	H	H	H	H	H	H	H	H	H

A represents a resistant phenotype or genotype; B represents a susceptible phenotype or genotype; H indicates heterozygous phenotype or genotype. The genotype of recombinant F_2:3_ families are shown in bold.

**Table 3:** Protein sequence comparison of pmXMM with the known Pm4-resistant alleles. Amino acid sequences of Pm4d and Pm4h were identified from the study of Sánchez-Martín et al. (2021).

Pm4 Protein Isoforms	Pm4_V1 Variance			Pm4_V2 Variance
Amino acid sites	205	208	395	713
pmXMM	E	L	T	A
Pm4a	K	W	T	A
Pm4b	E	L	T	G
Pm4c	E	L	T	G
Pm4d	E	L	T	A
Pm4e	E	L	T	A
Pm4h	K	L	A	A

## Data Availability

No applicable.

## Supplementary Materials

The following supporting information can be downloaded at: https://www.mdpi.com/article/10.3390/ijms23031194/s1.
